# Advancing Student Health and Achievement Through Medicaid: Lessons Learned from State Efforts to Expand Medicaid‐Funded School Health Services

**DOI:** 10.1111/josh.12969

**Published:** 2020-11-12

**Authors:** Alexandra Mays

**Affiliations:** ^1^ Healthy Schools Campaign 2545 W. Diversey Ave. ‐ Suite 214 Chicago IL 60647 USA

**Keywords:** school health services, health policy, school health policy, child and adolescent health, school funding

## Abstract

**BACKGROUND:**

School health services improve health and academic outcomes; however, sustainable funding for these services is an ongoing struggle. In December 2014, the Centers for Medicare & Medicaid Services clarified how Medicaid will reimburse school health services. School districts, once restricted to reimbursement for services delivered under specific conditions, can now receive Medicaid reimbursement for eligible services delivered to all Medicaid‐enrolled students.

**METHODS:**

This article examines the literature exploring school health services' impact on health and academic outcomes and Medicaid's role in funding school health services. The article analyzes state, school‐based Medicaid policies and programs and the impact of the federal policy change.

**RESULTS:**

As of August 2020, 13 states have used the federal policy change to expand their school‐based Medicaid programs to include all eligible services delivered to all Medicaid‐enrolled students.

**CONCLUSIONS:**

This policy change creates an opportunity for states and school districts to leverage health care funding to implement multiple components of the Whole School, Whole Community, Whole Child model, including health services, counseling, psychological and social services, employee wellness, and school climate. The federal policy change can also improve health equity by increasing reimbursement for school districts serving higher percentages of Medicaid‐enrolled students.

Research shows that access to school health services improves health and academic outcomes, particularly for students with chronic health issues; however, finding sustainable funding has been an ongoing struggle.^1^, ^2^

In December 2014, the Centers for Medicare & Medicaid Services (CMS) clarified the way that Medicaid will reimburse for health services delivered in schools.^3^ School districts, once restricted to reimbursement for services delivered to students enrolled in Medicaid under specific conditions, are now permitted to cover eligible services delivered to all Medicaid‐enrolled students. Put simply, this means more health care funding for the most disadvantaged students.

Even though the federal policy change opened the door to greater financial support for states and school districts, most states did not immediately take advantage of it. Many states had codified the original CMS policy, stating that districts could only seek reimbursement for health services delivered under a student's Individualized Education Plan (IEP) or Individualized Family Service Plan (IFSP).^4^, ^5^ Several had formalized the original policy in state law. It took time for early adopter states to develop implementation roadmaps for other states to follow.

Six years later, 13 states—California, Colorado, Connecticut, Florida, Kentucky, Louisiana, Massachusetts, Michigan, Missouri, Nevada, New Hampshire, North Carolina, and South Carolina—have successfully expanded their school‐based Medicaid programs, with more states working to do so.^6^ CMS, and many states, recognize this policy change as an opportunity to support student mental health.^7^

The policy change allows school districts to expand their school‐based Medicaid programs to cover more students and bring in additional, sustainable federal funding. It can also improve health equity by supporting increased reimbursement for school districts that serve higher percentages of Medicaid‐enrolled students.

Finally, it presents an important opportunity to support implementation of multiple Whole School, Whole Community, Whole Child (WSCC) components including health services, counseling, psychological and social services, employee wellness, and social and emotional school climate.

However, CMS presented an opportunity, not a mandate. There is a critical need to support states in implementing the policy change and ensure this opportunity is leveraged to expand access to and resources for school health services for children across the country.

## The Case for School‐Based Medicaid Expansion

Across the country, chronic and acute physical and mental health issues affect children's ability to succeed in the classroom.^8^ Nearly 1 in 10 children ages 5–19 are diagnosed with asthma and approximately 1 in 5 children ages 5–11 have at least 1 untreated decayed tooth.^9^, ^10^ In addition, 1 in 6 youth aged 6–17 experience a mental health disorder each year, such as depression or anxiety. ^11^ Left untreated or undermanaged, health issues can adversely affect children's attendance, their ability to see, hear, and pay attention in the classroom, and their chances of graduating from high school.^8^

Students in underserved communities, particularly students of color, are at increased risk of chronic health problems, such as diabetes and asthma, that hinder learning and impact long‐term health.^1^, ^8^ Ignoring these health inequities will undermine efforts to close the opportunity gap.^8^

No school district is immune. Across the country, a rising trend in youth suicides, anxiety and depression underscores the need for more behavioral and mental health services.^12^ School safety and school climate are also top concerns for educators and policymakers across the country and school health services, particularly mental health services, can play a critical role in addressing both.^13^

Schools are increasingly seen as places to deliver high‐quality, cost‐effective health care. Numerous studies show that access to school nurses and other school health providers can improve health and reduce absenteeism, particularly for students with chronic health issues.^14^, ^15^ Increased access to school health services can also improve care coordination and reduce health care costs.^15^

Yet more than half of public schools do not have a full‐time school nurse or school counselor, and only 13% of the nation's school‐age youth have access to services through a school‐based health center.^16^, ^17^ Schools in low‐income districts generally have lower nurse‐to‐student ratios and access to fewer health services than schools in wealthier districts.^18^

Finally, increasing access to school health services and programs, particularly mental health services and programs, is a proven strategy for supporting staff health and wellness and providing staff with safe and supportive working environments.^19^

## Opportunity Presented by CMS Policy Change

In 2014, CMS issued a letter to state Medicaid directors that clarified which services can be reimbursed by Medicaid in a school‐based setting.^3^

CMS' previous policy, called the free care policy, prohibited reimbursement for services provided to Medicaid‐enrolled students if those services were provided free of charge to all students.^5^ There were some exceptions: services could be submitted for Medicaid reimbursement if they were included in a student's IEP or IFSP or delivered through the Maternal and Child Health Block grant.^5^

The letter stated that schools can seek reimbursement for covered services provided to *all* students enrolled in Medicaid, regardless of whether the services are provided at no cost to other students. Table 1 includes examples of school‐based health services Medicaid might pay for in your state.

**Table 1:** Examples of school‐based health services Medicaid might pay for in your state

Mental health services Substance use services Oral health services Assessments (e.g. psychological status, health, nutrition, audiological) Occupational therapy Physical therapy Personal care services Speech, hearing and language services Vision screenings and services

The specific, reimbursable services in your state will be determined by the state Medicaid plan.

Expanding billing for more students—as well as expanding the types of services and providers being reimbursed—means more federal reimbursement to states and districts. And since most schools already deliver some of these services, bringing in federal reimbursement can replace scarce education money and help stretch resources further.^20^ Increased federal reimbursement helps ensure ongoing investment in and support for the delivery of school health services and ultimately help schools expand the staffing needed to provide physical and behavioral health services.

While the CMS policy change presents a tremendous opportunity to expand access to school health services through Medicaid reimbursement, work must be done at the state and district levels to take advantage of it.

## How States can Align with CMS Policy Change

In keeping with the previous CMS policy, many state Medicaid plans explicitly state that school districts may only seek reimbursement from Medicaid for health services delivered under a student's IEP or IFSP.^4^ In addition, several states formalized the policy in state law. For these states to leverage this opportunity to expand their school Medicaid programs, they must first remove related restrictions in their state Medicaid plan and state statute and then update their state's school Medicaid guidance.

Making these changes takes planning and organization, but change is possible: state Medicaid plans are intended to be living documents, and states frequently submit and receive approval for state plan amendments (SPA).^21^

Today, numerous issues—including student mental health needs, the opioid epidemic and school safety concerns—are driving momentum for states to leverage the opportunity to expand school health services. The early adopters that received approval to move forward with this change are already proving the importance and value of this effort.

As of October 2020, 13 states have used the policy change to expand their school‐based Medicaid programs to include all eligible services delivered to all Medicaid‐enrolled students.^6^ For example, as a result of this change, school districts in Louisiana and Nevada can now receive Medicaid reimbursement for all medically necessary services included in Medicaid's comprehensive Early, Periodic, Screening, Diagnosis and Treatment (EPSDT) benefit. In addition, a number of states, including Colorado and Michigan, added additional qualified providers, including school psychologists and behavior analysts, to their state Medicaid plan. As a result, school districts in Colorado and Michigan can now seek Medicaid reimbursement for services provided to Medicaid‐enrolled students by these providers.

Preliminary data indicates that leveraging this opportunity to expand school Medicaid programs results in a significant increase in federal Medicaid revenue to states.^22^ This revenue can then be used to hire additional school health providers, deliver additional services and ensure the sustainability of school health service programs in general.

## IMPLICATIONS FOR SCHOOL HEALTH

The CMS policy change creates a tremendous opportunity to increase funding for the most disadvantaged students, support implementation of the WSCC model and ultimately improve student health and academic outcomes. Efforts to date have led to important lessons that can inform the work of state and local leaders ready to explore what it will take to implement this opportunity.

### Leveraging Lessons Learned at the Local Level

For individuals working at the local level, consider the following action steps to advance implementation.

- *Build a team*. Consider who in the school or community (eg, school health providers, local pediatricians, local public health agency, advocates, and other stakeholders working to implement components of the WSCC model) will support increased access to school health services. Work with these individuals to assess opportunities and develop an action plan for strengthening and expanding school health services.
- *Assess the current school health services environment*. Gather the data needed to understand the school health services current being delivered and billed for in the school district, the providers delivering school health services, unmet student health needs and the opportunities for improvement in the school district or school.
- *Make the case*. Work with the team to share the data and action steps with key decision makers in the state. Engage the individuals who oversee the state school Medicaid program for the state education agency and state Medicaid agency, health and education staff in the governor's office and key state organizations, such as the state school nurses association and state parent teachers association. Consider collaborating with other school districts in the state who are also committed to expanding their school health services program to make the case to state leadership.
- *Be a champion*. Educate stakeholders from education, health care and public health sectors about the opportunity and how expansion of school health services and overall implementation of the WSCC model can support their mutual goal of ensuring students thrive.

### Leveraging Lessons Learned at the State Level

Once state decision makers are committed to moving forward with the opportunity to expand the state school Medicaid program, consider utilizing the following lessons learned to advance this work:

- *Collaborate across sectors*. A number of states leveraged existing teams or established new teams to bring together key people in the state education agencies, state Medicaid agencies and school districts.
- *Collect data*. Multiple states have conducted needs assessments to better understand the delivery of school health services and student health needs across the state. Data can be used to identify additional services and providers that the state might consider adding to their state Medicaid plan and inform how additional resources might be utilized to support implementation of the WSCC model.
- *Align efforts with health care transformation*. It is critical to understand what efforts are taking place in a state to improve the delivery of health care services and make the case for why the delivery of school health services can support this work.
- *Create a positive policy environment*. A positive policy environment includes policies that facilitate enrollment of eligible children in Medicaid and the Children's Health Insurance Program (CHIP), allows school health providers to bill Medicaid for eligible services, and ensures revenue from school‐based Medicaid programs is reinvested in school health programs at the local level. A positive policy environment also includes creating policies that support implementation of the WSCC model.
- *Leverage existing assets*. States can leverage their existing infrastructure and capacity to expand school Medicaid programs. It is key to remember that all states, except Wyoming, have existing school Medicaid programs providing a foundation upon which this expansion can occur.

Leveraging these lessons learned to implement the CMS policy change—and the opportunity it presents to increase access to and resources for school health services—offers both the health and education sectors a practical way to increase the role of schools in meeting the health needs of the nation's most vulnerable children and support implementation of the WSCC model.

### Human Approval Statement Subjects

Not applicable.

### Conflict of Interest

In the past 3 years, Healthy Schools Campaign has received grants from the following entities in support of the work that led to the findings shared above:

- Association of State and Territorial Health Officials: 2018—$180,000; 2019—$150,000; 2020—$150,000
- William & Flora Hewlett Foundation: 2019—$250,000; 2020—$175,000
- Kaiser Permanente: 2018—$100,000; 2019—$120,000; 2020—$130,000
- Robert Wood Johnson Foundation: 2018—$500,000
- W.K. Kellogg Foundation: 2018—$250,000

Whereas the author did not receive any direct funds from these entities, the funding was used by Healthy Schools Campaign to pay the author's salary.
